# The simultaneous crises of dengue and COVID-19 in Pakistan: a double hazard for the country’s debilitated healthcare system

**DOI:** 10.1186/s41182-022-00410-x

**Published:** 2022-02-25

**Authors:** Govinda Khatri, Mohammad Mehedi Hasan, Somina Shaikh, Syeda Lamiya Mir, Abdul Moiz Sahito, Priya, Ian Christopher N. Rocha, Osman Kamal Osman Elmahi

**Affiliations:** 1 Dow University of Health Sciences, Karachi, Pakistan; 2 Department of Biochemistry and Molecular Biology, Faculty of Life Science, Mawlana Bhashani Science and Technology University, Tangail, Bangladesh; 3 Division of Infectious Diseases, The Red-Green Research Centre, BICCB, Dhaka, Bangladesh; 4 School of Medicine, Centro Escolar University, Manila, Philippines; 5 Faculty of Medicine, Ibn Sina University, Khartoum, Sudan

**Keywords:** Dengue, COVID-19, Public health, Pakistan

## Abstract

Dengue fever is an arthropod-borne viral illness caused by four dengue virus serotypes (DENV 1–4), spread by Aedes mosquitos. Common symptoms of dengue include high peak temperature, headache, myalgia, and malaise following infection, with a rash emerging after 3 to 4 days. More than half of the world's population lives in dengue-endemic areas. Every year, around 100 million dengue cases are reported, with Southeast Asia comprising the majority. Nearly every day, there is a breakout of dengue infections in many areas of Pakistan, in addition to the ongoing COVID-19 pandemic. As a result, combating the dual burden of dengue and COVID-19 is difficult for the nation's healthcare system. Misdiagnosis owing to overlapping symptoms with COVID-19, overburdening of the healthcare system, and a lack of appropriate vaccination are some of the obstacles for dengue infection management. The government of Pakistan is pursuing a variety of measures to combat dengue fever outbreaks, including, The Pakistan Red Crescent Society was asked by the Department of Malaria Control Program to aid in promoting awareness and organizing clean-up campaigns in polluted regions and stagnant water for vector control.

## Introduction

Dengue fever is an arthropod-borne viral disease caused by four serotypes of dengue virus (DENV 1–4), spread by *Aedes* mosquitos. Dengue virus infection can cause a variety of symptoms; however, most infections are asymptomatic or subclinical [1]. Following the beginning of infection, the signs of the DENV include high peak fever, headache, nausea, vomiting, malaise, and myalgia, with a rash appearing after 3 to 14 days [2]. The critical phase of dengue may also present with warning signs, such as abdominal pain or tenderness, persistent vomiting, clinical signs of capillary leakage, loss of fluid from the intravascular compartment, lethargy or restlessness, mucosal bleeding, hepatomegaly, increased hematocrit, and thrombocytopenia. In addition, patients with severe dengue infection may sometimes show bleeding manifestations (dengue haemorrhagic fever) and shock (dengue shock syndrome) [3]. There is no specific anti-viral drug for dengue fever; however, proper fluid management and pain medications, including acetaminophen, are advised to be used, while aspirin should be avoided, since it might exacerbate bleeding [3]. Dengvaxia, a vaccination for persons aged 9 to 45, is available in several countries; according to the World Health Organization (WHO), the vaccine can be administered in patients with confirmed prior dengue infection [4].

More than half of the world's population lives in dengue-endemic areas. Every year, around 100 million dengue cases occur, with Southeast Asia having the largest burden [5], followed by Latin America [1]. According to the WHO, the first documented incidence of dengue fever in Pakistan occurred in 1994, although the yearly epidemic pattern began in Karachi in November 2005. Before 2006, dengue was limited to a few areas of Pakistan. Since 2010, Pakistan has been plagued by annual dengue outbreaks, which peak in the post-monsoon season [6]. The present fast spread of dengue to additional areas of Pakistan is due to *Aedes* mosquitoes moving from dengue-infected areas to unaffected areas, as well as vertical transmission [7]. Pakistan is still dealing with the dengue fever crisis in 2021. On September 2, 2021, 690 cases were detected across Punjab, bringing the overall number to 1082 in Punjab, 800 cases of dengue were reported in Khyber Pakhtunkhwa, and 32 people were infected in the federal capital Islamabad [8]. However, the exact number of cases in the country is uncertain due to inconclusive information in the media, diagnostic discrepancies, and rapidly rising cases each day.

Currently, Pakistan, such as the rest of the world, is grappling with the COVID-19 crisis, which has overwhelmed Pakistan's health system due to infrastructure and the country's weak economy, and another crisis might lead to the demise of the health care sector [9]. Along with COVID-19, mucormycosis infection is also a threat to Pakistan [10], hence aggressive efforts to combat the spread of such heinous diseases are required.

The aim of this article is to assess the current state of dengue in Pakistan to better understand the existing efforts, burden, and difficulties in controlling the dramatic rise in dengue cases amid the ongoing COVID-19 crises, as well as to identify opportunities for development through advanced proposals for all health care workers and the authorities.

## Burden and current status of dengue in Pakistan

Dengue has been a threat in Pakistan for a long time with many cases of the virus occurring all over country. Dengue fever has been widespread in Pakistan's southern coastal city Karachi for decades, but significant epidemics in the northeast have only erupted around 9 to 10 years earlier. Dengue virus incidences still continue to develop in various locations of Pakistan beginning in January 2021 [11].

Pakistan's climate is warm and moist, especially in the major cities of Karachi and Lahore. Unplanned urbanization has resulted from the dramatic population growth, leading to an increase in sanitary issues. Open or blocked sewers, stagnant water, and waste dumps can be seen in abundance. The overuse of water (for example, to wash cars, gardens, verandas, grounds, and roads), causes water to sit still for days. These conditions contribute to higher *Aedes* mosquito breeding rates. These mosquitoes, which used to reside in the woods, became domesticated as a result of extensive travel and trade around the world [12]. Aside from that, dengue virus epidemics are associated with socioeconomic factors, viral evolution, and population increase [13]. Thousands of dengue infections occur in Pakistan each year, with a relatively high number of cases in 2019. The dengue virus is rapidly spreading, particularly in federal provinces, with hundreds of cases reported every day, and this could lead to a serious health crisis, such as COVID-19.

From 1 January to 25 November 2021, a total of 48,906 dengue cases were reported, with 183 deaths (case fatality ratio (CFR): 0.4%) in 4 provinces, Islamabad and Azad Jammu and Kashmir autonomous territories, Pakistan. Punjab province had the most instances, having 24,146 cases and 127 fatalities (CFR: 0.5 percent) as of November 25, accounting for 49.4 percent and 69.4 percent of all cases and deaths, correspondingly. The majority of the deaths were recorded in the Lahore district [8].

### Challenges

The COVID-19 pandemic has a larger impact on the torrid zone of the world than on cold zones, where dengue disease, caused by the DENV, is already common. Doctors should be aware of the possibility of dengue and COVID-19 coinfection, as well as a potentially dangerous interaction between them, in areas, where outbreaks overlap [14]. According to WHO, there were 1,393,887 confirmed cases of COVID-19 in Pakistan from 3^rd^ January 2020 to 28^th^ January 2022 with 29,162 fatalities [15].

The following are the challenges that might limit the response capacity:

#### Mutual symptoms leading to misdiagnosis

Symptoms of these illnesses are identical and overlap in the acute periods, which may hinder adequate diagnosis and management. Patients described complaints of fever, fatigue, muscle aches, skin rashes, and petechiae with normal chest radiograph, reflecting several viral conditions, making it more challenging to differentiate between dengue and COVID-19 [16].

#### Overburdened healthcare system

Due to the ongoing COVID-19 pandemic, all the facilities of health care are directed towards it which may cause the dengue cases to be underreported. It can be due to the exhaustion of specialized beds, medications, laboratory equipment for virologic surveillance, and experienced doctors in dengue [6]. Due to a spike in coronavirus infections across the province, about 60% of beds allocated for coronavirus patients were filled in April 2021. Whereas an official reported 75 to 80 percent beds occupancy of ICU beds and private hospitals no longer admitting patients requiring ventilator [17]. According to a newspaper in May 2020, 163 high dependency units out of 174 for COVID-19 patients were occupied in Karachi [18].

#### Lack of proper vaccine

Sanofi-Pasteur produced a tetravalent dengue vaccine that has been authorised for clinical use in a number of endemic regions, but its performance in the general population has been called into question. The treatment is made by limiting the symptoms that appear during infection. The serious issue is that Pakistan's healthcare system lacks the funding and capacity to conduct research to develop effective vaccinations to protect the vulnerable population. Moreover, vaccination is also required for prevention of COVID-19 pandemic, only 36.8% of population of Pakistan is vaccinated [19].

### Prevention and control of COVID-19

COVID-19 can be prevented through the following steps.

- Avoiding congested environments, enclosed spaces with poor ventilation, and prolonged social engagement.
- Avoid touching objects, particularly in public areas or medical facilities. Decontaminate surfaces routinely with standard disinfectants.
- Hands should be washed often with soap and water or with a hand sanitizer. Always have an alcohol-based hand sanitizer and apply it routinely.
- Wear mask and avoid shaking hands.
- Maintain a safe distance and a minimum of one meter between yourself and others, even if they do not appear to be unwell, because the virus can infect people who do not show any symptoms.
- Get vaccinated.

Government of Pakistan has taken various preventive and control measures to limit the SARS-COV-2 virus, such as establishment of Quarantine centres, lockdown, implementation of SOPs, awareness campaigns, travel restrictions, and production of ventilators [20].

### Efforts

There were just 14,000 beds available in the provinces of Lahore, Karachi, and southern Sindh, which accounted for roughly 68,000 of the country's 98 thousand patients. Many people had contacted hospital administrations, but hospitals failed to give adequate medical attention given the shortage of medical resources. Nurses were stressed, testing facilities were overburdened, and emergency rooms were overflowing with sick patients. Similarly, the staff has shrunk, ICUs have run out of room, and cost for the treatment has risen [21]. To supplement standard surveillance and response efforts, community-driven interventions have long been a top priority in combating issues, such as COVID-19 and dengue.

1. The Ministry of Health's Field Epidemiology and Disease surveillance division is striving to enhance dengue monitoring, case management, and outbreak intervention at primary health care centers and hospitals. These include arranging clean-up drives at the polluted areas and stagnant water for vector control and setting up logistic support, such as insecticides, medicine, rapid diagnosing tests, for dengue control [22].
2. The Department of Malaria Control Program approached the Pakistan Red Crescent Society to aid in raising awareness, providing mosquito nets to isolate confirmed and suspected patients to limit additional transmission, and protecting disadvantaged groups from mosquito bites during the day [23].
3. The federal government established dengue epidemic response group at district and tehsil (administrative division of district) level which check residences and spray insecticides to control the larvae and restrict the mature insect population [24].
4. Public awareness efforts were also taken to educate the public and assist them in taking the necessary actions to protect themselves from dengue fever [3].
5. In Pakistan, the National Institute of Health created an Android-based programme called Mosquitoes Alert to help people learn about the many types of mosquitos in their region and the illnesses they might cause [23].

Although these efforts are admirable, the outcomes have been disappointing, with cases increasing each year during the monsoon season and the vulnerable people left at the mercy of such a dreadful illness.

### Recommendations

Amidst the burden of the COVID-19 pandemic, well-known cities, such as Karachi, Islamabad, and Lahore face the challenge of widespread endemic disease, such as dengue. The employment of precise and effective measures to combat the devastation caused by dengue fever is urgently needed. The following are some essential parameters for successful dengue management during and apart from health emergencies that planners and policymakers should consider:

- Health officials should identify potential vector breeding grounds and work on interventions to drain them as early as possible. Eliminating superfluous containers that hold water in which *Aedes* mosquito may deposit its egg is one feasible and suggested environmental management method. Source reduction is the terminology for this method. Mosquitoes get limited chances to deposit its egg and cannot grow through their aquatic developmental periods when pot shelters are eliminated and containers that hold water are wrapped in a fine mesh to keep mosquitoes away. When done on a regular basis, source reduction may be quite successful.
- Dengue surveillance should be integrated into the nationwide healthcare information system, with a range of key indicators monitored at all levels of the healthcare. Record should be kept about quantity of dengue cases, severe dengue cases and deaths related to dengue. System of reporting potential risk indicators of dengue should also be improved like mosquito breeding sites and effective measures should be taken to eliminate them by cleaning them and fitting water filled containers with lids and other biological measures should be taken as well.
- Multisectoral dengue action committee is recommended to be established and an emergency action plan needs to be devised for outbreak preparedness.
- They should also educate the public on how to protect themselves from mosquito bites using strategies, such as mosquito nets, insect repellents, and covered clothing, as well as actively intervene to control vector populations by ensuring proper waste disposal and regular maintenance of public places.
- Government authorities should prioritize funding and support to improvise sanitation and sewage systems especially in urban areas, where sewage disposal is often overlooked.
- Municipal authorities must be vigilant during rainy seasons to prevent rainwater from accumulating and provide breeding grounds for the vector.
- As for COVID-19, frequent testing should be made accessible for the symptomatic cases and isolation facilities should be provided to prevent the spread of the infection, and aggressive contact tracing should be promoted to advise quarantine to those who may have been infected from the individual.
- Mutations in the DENV serotype have contributed to the spread of dengue during the pandemic. To combat this, continued surveillance for dengue fever is essential.
- Hospitals should increase their accessibility for dengue patients by creating more space and resources for prompt treatments.
- Due to overlapping symptoms of dengue and COVID-19, such as fever, rash, headache, and respiratory problems, the possibility of co-infections and false-positive dengue serology is raised. There is an increasing need for rapid tests capable of distinguishing SARS‐CoV‐2 and DENV with higher sensitivity. Laboratory testing facilities should bring in newer testing technologies, such as antibodies testing and polymerase chain reaction.

## Conclusions

As the article analyses the existing potential pitfalls, circumstances, as well as harm caused by the COVID-19 pandemic to the Dengue-affected community, an inference is reached that addresses the most important suggestions for future improvement. The article highlights the unseen problems and obstacles that the people of Pakistan confront. Authorities and the general public must be vigilant at this critical juncture in world history to prevent the dengue virus outspread. Dengue fever has become more dangerous as a result of the precarious situation surrounding the COVID-19 pandemic. Pakistan's healthcare system is in disarray. With 0.6 beds for every 1000 people and health spending less than 0.75 percentage of GDP, it is difficult for the Pakistan's healthcare system to resist the COVID-19 impact amid the occurrence of an influx of new cases. With successful methods, public awareness, and public cooperation, the government can still eradicate the dengue virus from its area.

## Data Availability

Not applicable.

## Abbreviations

COVID-19: Coronavirus disease 2019
DENV: Dengue virus
WHO: World Health Organization
GDP: Gross Domestic Product
CFR: Case fatality ratio
ICU: Intensive Care Unit
SOP: Standard Operating Procedure
